# miR-155-3p in Systemic Lupus Erythematosus: Association with Immune Activation and Renal-Related Parameters

**DOI:** 10.1007/s12010-026-05663-4

**Published:** 2026-04-11

**Authors:** Mohamed M. Sadaty, Rana Mohamed, Emad El-Zayat, Salma M. Mekhemer, Amira El-Ansary, Nahla O. Mousa

**Affiliations:** 1 Department of Medical Laboratory Technology, Faculty of Applied Health Science Technology, Misr University for Science and Technology, Giza, 3237101 Egypt; 2 Department of Biotechnology, Faculty of Science, Cairo University, Giza, 12613 Egypt; 3 Department of Internal Medicine, Faculty of Medicine, Misr University for Science and Technology, Giza, 3237101 Egypt

**Keywords:** Systemic Lupus Erythematosus (SLE), Diagnostic Biomarkers, MiR-21-5p, MiR-155-3p

## Abstract

Systemic lupus erythematosus (SLE) is an autoimmune illness that affects multiple organs, causes hematological abnormalities, and alters immunological regulation. Identifying reliable biomarkers is critical for improving the diagnosis and monitoring of disease activity. This study aimed to evaluate clinical, biochemical, immunological, and molecular alterations in SLE patients compared with healthy controls, with a focus on the exploratory discrimination of whole-blood miRNA expression and conventional serological markers. A total of 100 SLE patients and 50 age-and gender-matched healthy controls were evaluated. Hematological, biochemical, and immunological markers were examined with miR-21-5p and miR-155-3p expression. Clinical relevance was determined using correlation analyses and ROC curve evaluations. SLE patients had significantly lower hemoglobin, WBCs, and platelets compared to controls (*p* < 0.001). Inflammatory indicators, such as CRP and ESR, were significantly higher (*p* < 0.001). Immunological testing showed significant increases in ANA, anti-dsDNA, and anti-Smith antibodies, as well as decreased complement (C3, C4) levels (all *p* < 0.001). miR-155-3p was considerably elevated in SLE patients and linked with anti-Smith antibodies and creatinine (*p* < 0.05), but miR-21-5p had no diagnostic significance. ROC analysis showed excellent diagnostic performance for ANA (AUC = 0.992) and anti-dsDNA (AUC = 0.973), with good accuracy for miR-155-3p (AUC = 0.813, *p* < 0.001). SLE patients have significant hematological, immunological, and renal changes. Among molecular indicators, miR-155-3p is associated with immune activation and selected renal-related laboratory parameters, including serum creatinine, without implying definitive renal involvement, indicating its potential as a complementary biomarker. These findings suggest that miR-155-3p may serve as an exploratory complementary biomarker alongside conventional serological markers; however, its incremental diagnostic value requires validation using multivariable diagnostic models in prospective studies.

## Introduction

Systemic lupus erythematosus (SLE) is a chronic, inflammatory, multisystem autoimmune disease that affects mostly women of reproductive age [1]. Current research suggests that SLE develops through a complex disturbance stemming from an interaction between environmental exposures, hormonal changes, genetic background, and epigenetic influences, together with other unknown factors. SLE is distinguished by a dysregulated immune response through overactivated dendritic cells (DCs), T-cells, aberrant B-cell activation, and impaired clearance of apoptotic cells, which together result in the development of a wide array of autoantibodies, such as anti-nuclear and antiphospholipid antibodies leading to immune complex formation and deposition in tissues, causing damage to multiple organs such as kidneys, Skin, joints, heart, and central nervous system [2]. The disease’s erratic course is characterized by flares and remissions, along with its broad and often nonspecific spectrum of clinical manifestations ranging from mild cutaneous lesions to life-threatening organ involvement, makes early identification and accurate diagnosis particularly challenging, especially in the initial stages when symptoms may overlap with other autoimmune or inflammatory disorders [3].

Beyond its complex pathophysiology, SLE imposes a significant health and socioeconomic impact on the world. Chronic weariness, discomfort, and organ dysfunction can have a negative impact on patients’ quality of life. Furthermore, SLE disproportionately affects women during their reproductive years, resulting in high morbidity and healthcare expenses. Despite improvements in recent decades, mortality rates remain higher than those of the general population, especially due to cardiovascular problems, infections, and renal involvement [4].

Many classification and diagnostic criteria have been created to help clinicians identify SLE cases. The American College of Rheumatology (ACR) and Systemic Lupus International Collaborating Clinics (SLICC) criteria are the most utilized. These are based on clinical symptoms and laboratory markers, including antinuclear antibodies (ANA), anti-double- stranded-DNA (anti-dsDNA), and decreased complement levels (C3, C4). However, these markers possess notable limitations. For instance, ANA positivity is not exclusive to SLE and is associated with several autoimmune diseases, while complement levels can fluctuate due to infections. Furthermore, many of these indicators are insufficiently sensitive or specific to confirm or rule out SLE, particularly in early disease stages or with atypical manifestations [5].

Although standard criteria have been modified throughout time, they frequently fail to identify patients in the early stages of disease or those who exhibit unusual symptoms. Proteomics, metabolomics, and transcriptomics are emerging technologies that have been studied to supplement traditional assays. However, these approaches are expensive, time-consuming, and not generally used in ordinary clinical settings. Hence, the challenge remains in identifying accurate, accessible biomarkers that might bridge the gap between research findings and real-world clinical use [6]. The ideal biomarker would be easily accessible, non-invasive, and capable of reflecting underlying immunological changes specific to SLE. Recent advancements in molecular biology and genomics have opened new pathways for biomarker discovery, one of which is microRNAs [7].

miRNAs are small single-stranded non-coding RNA molecules (about 22 nucleotides) that regulate gene expression at the post-transcriptional level. MicroRNAs perform critical roles in a variety of biological processes, including cell differentiation, proliferation, and apoptosis, and play a crucial role in regulating the function and activity of both innate and adaptive immune cells. Importantly, miRNAs are highly persistent in bodily fluids like plasma, serum, and urine, making them promising potential biomarkers for use as circulating biomarkers. Their stability arises from their packing within exosomes or attachment to RNA-binding proteins, which protects them from breakdown by RNases in the circulation [8].

Recent research has revealed that miRNAs are differentially expressed in autoimmune disorders, including SLE, and may be implicated in disease etiology. In 2007, Dai et al. reported distinct differences in microRNA (miRNA) expression profiles between patients with systemic lupus erythematosus (SLE) and healthy controls. Their study identified seven miRNAs that were downregulated (miR-196a, miR-L7-5p, miR-409-3p, miR-141, miR-383, miR-112, and miR-184) and nine that were upregulated (miR-189, miR-61, miR-78, miR-21, miR-142-3p, miR-342, miR-299-3p, miR-198, and miR-298). These findings suggest that alterations in miRNA expression may serve as potential diagnostic biomarkers for SLE and play a significant role in the disease’s underlying pathogenesis. Some miRNAs are known to affect immune cell differentiation, cytokine signaling, and inflammation, all of which play important roles in the development of lupus. Among the miRNAs implicated in autoimmune regulation, miR-155-3p and miR-21-5p have received significant focus [9].

MiRNAs have a significant impact on immune regulation because they modulate pathways such as T cell receptor signaling, B cell maturation, cytokine release, and death. Dysregulation of miRNAs undermines immunological tolerance, increases autoreactive cells, and maintains chronic inflammation—all hallmarks of SLE pathogenesis. This immunological role exposes miRNAs not just as indicators but also as potential therapeutic targets, capable of restoring immune homeostasis when appropriately managed [10].

Among these, miR-155 plays a vital function in both innate and adaptive immunity. miR-155 is overexpressed in inflammatory autoimmune illnesses including rheumatoid arthritis and systemic lupus erythematosus. Functional studies indicate that miR-155 is involved in the development of several disorders. Human and animal models of many autoimmune illnesses, including rheumatoid arthritis (RA), multiple sclerosis (MS), systemic sclerosis (SSc), and systemic lupus erythematosus (SLE), were found to have altered miR-155 expression [11].

In addition, miR-21-5p controls gene expression during T cell activation. As a result, its higher expression in SLE patients is closely related to disease activity. miR-21-5p has been associated with T-cell differentiation and immune response regulation [12].

miR-21-5p has emerged as one of the most often dysregulated microRNAs in systemic lupus erythematosus (SLE). Its expression levels are markedly higher in patients with active disease, and there is a clear link with clinical and biochemical markers of disease activity. Elevated miRNA-21 contributes to immunological dysregulation by altering T cell responses and activating inflammatory pathways, both of which are key factors in SLE pathogenesis [13].

Despite these promising findings, the diagnostic value of whole-blood miRNA expression in SLE is still being investigated. Little research has rigorously investigated their diagnostic accuracy, and there is little information on their aggregate predictive value when compared. Furthermore, ROC curve analysis was used to explore the discriminatory performance of individual biomarkers [14].

However, most of the research looking into whole-blood miRNA expression in SLE has been restricted by small sample sizes, diverse patient demographics, or a lack of thorough statistical validation. Furthermore, most studies have examined single miRNAs in isolation, ignoring the possible additive effect of integrating numerous markers. This highlights the importance of well-designed studies that systematically test the diagnostic performance of potential miRNAs, both alone and in combination, to give greater evidence for their therapeutic value [15].

In the current study, we explored the expression of two selected miRNAs (miR-21-5p and miR-155-3p) in patients with established SLE compared with age- and sex-matched healthy controls. We aimed to evaluate their association with key immunological and renal-related laboratory parameters and to assess their exploratory discriminatory performance alongside conventional serological markers. Given the case-control cross-sectional design, the findings are hypothesis-generating and require validation in prospective diagnostic cohorts that include disease-control populations [16].

The identification of these miRNAs as diagnostic biomarkers has the potential to significantly impact SLE clinical care. Such biomarkers would not only help with early and accurate diagnosis, but they could also reduce the need for invasive procedures and allow for earlier treatment intervention. Furthermore, knowing miRNA expression patterns may help to improve knowledge of the molecular mechanisms underlying SLE and open new options for targeted therapy development.

## Materials and Methods

### Study Design and Ethical Approval

This was a case–control, cross-sectional study comparing established SLE cases with healthy controls. This study was conducted over a 12-month period, from January 2024 to January 2025, at the Rheumatology Unit of Memorial Souad Kafafi University Hospital. A total of 100 individuals (81 women and 19 men), aged between 15 and 60 years, who had been clinically diagnosed with systemic lupus erythematosus (SLE), were enrolled. Each diagnosis was carefully confirmed by experienced rheumatologists through detailed clinical assessment and supporting laboratory investigations, including serological, immunological, and hematological tests such as ANA, anti-dsDNA, CRP, ESR, C3, C4, and anti-Smith antibodies. Patients were classified according to the diagnostic criteria of the American College of Rheumatology (ACR) and the Systemic Lupus International Collaborating Clinics (SLICC). Renal involvement was defined a priori based on the presence of elevated serum creatinine and/or proteinuria above the laboratory reference range at the time of sampling, in the absence of other known causes of renal dysfunction. A group of healthy volunteers, matched for age and sex, was included as the control group. The control group consisted exclusively of healthy individuals; disease-control groups were not included in the present study. Patients with confirmed SLE according to ACR/SLICC criteria were included. Exclusion criteria included pregnancy, active infection at the time of sampling, malignancy, coexisting autoimmune diseases, diabetes mellitus, and recent vaccination or acute inflammatory conditions. Healthy controls had no history of autoimmune or inflammatory diseases. The study protocol was reviewed and approved by the Ethics Committee of Misr University for Science and Technology **(FWA00025577).** Written informed consent was obtained from all participants or their legal guardians. All procedures were conducted in accordance with institutional and international ethical standards.

Patient data were collected in three components. The first encompassed sociodemographic characteristics, including age, sex, residence, occupation, and smoking status. The second addressed clinical history, covering family history of autoimmune diseases, presenting symptoms, disease duration, and current treatment regimen. The third included laboratory findings, comprising biochemical, immunological, hematological, and molecular analyses.

### Sample Collection and Processing

Each participant provided a 5 mL venous blood sample, which was processed as follows. A portion of the sample was immediately analyzed for complete blood count (CBC), erythrocyte sedimentation rate (ESR), and glycated hemoglobin (HbA1c). The remaining blood was stored at − 80 °C until RNA extraction. Serum was separated by centrifugation of 3 mL of blood at 4000 × g for 10 min and was subsequently used for biochemical and immunological analyses, including alanine aminotransferase (SGPT), creatinine, lipid profile, antinuclear antibodies (ANA), anti-double-stranded DNA (anti-dsDNA), C-reactive protein (CRP), complement components C3 and C4, and anti-Smith antibodies.

## Immunological and Serological Assays

The serum level of anti-double-stranded DNA (anti-dsDNA) IgG was quantified using a commercially available ELISA kit (Pishtaz Teb Diagnostics, Cat. No. PT-dsDNAIgG-96). Antinuclear antibodies (ANA) were measured by ELISA following the manufacturer’s protocol (Pishtaz Teb Diagnostics, Cat. No. PT-ANA-SCIgG-96). C-reactive protein (CRP) concentrations were determined turbidimetrically using a fixed-time measurement method. Complement components C3 and C4 were assessed by radial immunodiffusion employing specific kits provided by FAR Diagnostics (Code 4405; Lot 91500 for C3 and Lot 85100 for C4). Anti-Smith antibodies were measured using an ELISA kit (ORGENTEC Diagnostika, 55129 Mainz – Germany), according to the manufacturer’s instructions.

## Hematological and Biochemical Analyses

Complete blood count (CBC) analysis was performed using an automated hematology analyzer (Sysmex, Japan). The erythrocyte sedimentation rate (ESR) was determined by the Westergren method and expressed in millimeters per hour (mm/h). Biochemical parameters, including alanine aminotransferase (ALT), serum creatinine, lipid profile (total cholesterol, triglycerides, high-density lipoprotein [HDL], and low-density lipoprotein [LDL]), as well as glycated hemoglobin (HbA1c), were measured using an automated clinical chemistry analyzer (Erba Mannheim XL-180, Germany). Urinary protein concentration was measured in a spot urine sample using an automated clinical chemistry analyzer according to the manufacturer’s instructions and expressed as mg/dL.

## miRNA Extraction and Expression Analysis

miRNA expression was assessed from total RNA extracted from EDTA-anticoagulated whole blood. using the QIAzol^®^ Lysis Reagent (QIAGEN, Germany, Cat. No. 217004). Extracted RNA was reverse transcribed into complementary DNA (cDNA) using the miScript II RT Kit (QIAGEN; Cat. No. 339320) Specific for hsa-miR-21-5p, hsa-miR-155-3p, and the endogenous reference hsa-miR-103a-3p on an Applied Biosystems real-time PCR system. SYBR^®^ Green-based miRNA assays (QIAGEN, Cat. No. 339306) were used to perform quantitative real-time PCR.

All reactions were run in technical duplicates under identical conditions. Ct values of the reference miRNA were examined across study groups and showed no significant differences, supporting its stability for normalization. Samples with failed amplification or undetermined Ct values were excluded from analysis. Relative expression was calculated using the 2^-ΔΔCt method, while log2 fold change (−ΔΔCt) was used for visualization. Validated miScript SYBR^®^ Green assays (QIAGEN) specific for hsa-miR-155-3p and hsa-miR-21-5p were used. These assays are designed to detect the mature miRNA strand and ensure strand specificity. The thermal cycling settings included an initial activation step at 95 °C for 2 min, followed by 40 cycles of denaturation at 95 °C for 10 s and a combined annealing/extension at 56 °C for one minute. After amplification, a melting curve analysis was performed over the range of 60–95 °C to ensure the specificity of the amplified products. RNA concentration and purity were assessed spectrophotometrically prior to reverse transcription. Negative controls were included in each run to exclude contamination and genomic DNA amplification. Inter-run calibration was performed using consistent assay conditions across plates.

The expression levels of circulating miR-21-5p and miR-155-3p were measured. 2^-ΔΔCt were normalized to the endogenous reference hsa-miR-103a-3p, the endogenous reference miRNA hsa-miR-103a-3p was selected based on previous reports demonstrating its stable expression in whole-blood samples and immune-mediated conditions. In the present cohort, 2^-ΔΔCt values of hsa-miR-103a-3p did not differ significantly between SLE patients and healthy controls, supporting its suitability for normalization. and relative expression was computed using the comparative ∆∆Ct method compared to the healthy control group. For visualization purposes, miRNA expression values are presented as log2 fold change (−ΔΔCt), which allows representation of both up- and down-regulation relative to the control group.

### Statistical analysis

Statistical analyses were performed using SPSS (version 26.0; IBM Corp., Chicago, IL, USA). Normality of continuous variables was assessed using the Shapiro–Wilk test. Normally distributed variables are presented as mean ± standard deviation (SD) and were compared using the independent samples t-test. Non-normally distributed variables are presented as median (interquartile range, Q1–Q3) and were compared using the Mann–Whitney U test. Correlations between miRNA expression levels and clinical or laboratory parameters were assessed using Spearman’s rank correlation coefficient. Receiver operating characteristic (ROC) curve analysis was used to evaluate the discriminatory performance of biomarkers between SLE patients and healthy controls. Optimal cut-off values were determined using the Youden index (maximum sensitivity + specificity − 1). For biomarkers that decrease in disease (e.g., complement C3 and C4), ROC directionality was defined accordingly. Given the exploratory case–control design and the evaluation of multiple biomarkers, no formal correction for multiple testing was applied; results are therefore interpreted as hypothesis-generating. A two-sided p value < 0.05 was considered statistically significant.

## Results

A total of 100 patients with established SLE and 50 healthy controls were included. Group comparisons of hematological, biochemical, immunological, and miRNA expression measures are presented below. The results were analyzed step by step, beginning with clinical and demographic features, then basic hematological and biochemical parameters, followed by inflammatory and immunological indices, and finally the evaluation of microRNA expression, their correlations, and diagnostic performance using ROC curve analysis.

## Clinical and Demographic Features

**Fig. 1:**
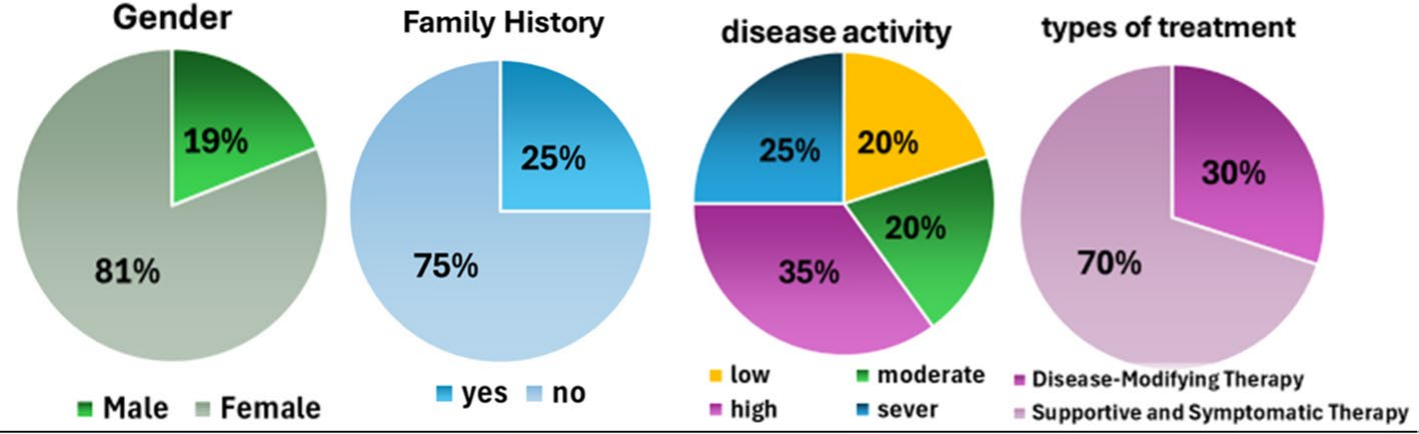
The pie chart illustrates the frequency distribution of gender, SLE disease activity, family history, and treatment type among SLE patients

Disease activity testing found that more than half of the cohort (60%) had high to severe disease activity, with 35% classified as high and 25% as severe, while only 40% fell into the moderate (20%) or low (20%) activity groups. This distribution demonstrates the substantial disease prevalence in this patient population. Disease activity was assessed at the time of sample collection using the Systemic Lupus Erythematosus Disease Activity Index (SLEDAI). Patients were categorized according to SLEDAI score as follows: low activity (SLEDAI ≤ 4), moderate activity (SLEDAI 5–9), high activity (SLEDAI 10–14), and severe activity (SLEDAI ≥ 15). Regarding therapy, 70% of patients received supportive and symptomatic care included low-dose corticosteroids and adjunctive therapies, whereas 30% Patients were receiving standard SLE therapies, including antimalarials (e.g., hydroxychloroquine), corticosteroids, and immunosuppressive agents such as azathioprine, mycophenolate mofetil, or cyclophosphamide, according to disease severity and clinical indication. Furthermore, 25% of patients reported a family history of autoimmune disease, indicating a potential genetic risk, but the majority (75%) did not, as indicated in Table 1 & Fig. 1.

**Table 1:** Baseline demographic characteristics of SLE patients and healthy controls

Parameters	Controls (*n* = 50)		SLE patients (*n* = 100)		Test		*p*-value
	Mean ± SD						
**Age**	**31.20 ± 8.53**		**35.82 ± 10.91**		**t-test= −2.839**		**0.005**
**Gender**	**Male = 15** **Female = 35**		**Male = 19** **Female = 81**		***X***^***2***^ **= 2.301**		**0.129**
**Frequencies and distribution of SLE patients regarding their socio- demographic and clinical characteristics**							
**Characteristics**		**Categories**		**N**		**%**	
Family History		Yes		25		25.0%	
		No		75		75.0%	
Disease activity		Low		20		20.0%	
		Moderate		20		20.0%	
		High		35		35.0%	
		Sever		25		25.0%	
Treatment types		Disease-Modifying Therapy		30		30.0%	
		Supportive and Symptomatic Therapy		70		70.0%	
Duration of disease categories		Less than 5 years		34		34.0%	
		Greater than or equal to 5 or less than 10 years		43		43.0%	
		Greater than 10 years		23		23.0%	

## Hematological and Biochemical Findings

An initial analysis of hematological indicators revealed significant disparities between the two study groups. Patients with SLE had significantly lower hemoglobin levels (9.74 ± 1.26 g/dl) than healthy controls (12.15 ± 1.30 g/dl; t = 10.91, *p* < 0.001). This observation is consistent with the anemia frequently observed in SLE patients, which could be caused by chronic inflammation, autoimmune hemolysis, or decreased erythropoiesis. Patients had a decreased mean red blood cell (RBC) count (4.22 ± 0.49 × 10⁶/µL) compared to controls (4.42 ± 0.43 × 10⁶/µL; *p* = 0.014), suggesting chronic disease-related anemia.

White blood cell (WBC) counts decreased significantly in SLE patients (4.51 ± 1.10 × 10³/µL) compared to controls (7.41 ± 1.68 × 10³/µL; *p* < 0.001). This leukopenia is a well-documented immunological characteristic of SLE, frequently associated with autoantibody-mediated destruction or bone marrow suppression. Platelet counts were significantly lower in patients (169.00 ± 56.87 × 10³/µL vs. 268.64 ± 65.05 × 10³/µL; *p* < 0.001), indicating thrombocytopenia linked to disease activity and autoantibody generation.

Interestingly, there was no significant difference in the percentages of neutrophils and lymphocytes across the groups (*p* > 0.05), implying that while total leukocyte numbers are reduced, the relative distribution of these immune cell subsets may stay unchanged.

Renal and liver function were also assessed. SLE patients had significantly higher serum creatinine levels (1.22 ± 0.56 mg/dL) compared to controls (0.93 ± 0.16 mg/dL; *p* < 0.001) and proteinuria (37.21 ± 35.60 mg/dL vs. 4.83 ± 2.78 mg/dL; *p* < 0.001). These findings indicate alterations in renal-related laboratory parameters, reflected by increased serum creatinine and urine protein levels., which is one of the most severe and clinically significant symptoms of SLE. In contrast, no significant differences were seen between the groups in ALT, cholesterol, triglycerides, or HbA1c, indicating that liver function and metabolic parameters were not significantly affected in this cohort.

### Inflammatory and Immunological Markers

Systemic inflammatory markers were significantly increased in SLE patients compared to controls. Patients had significantly higher CRP levels (18.87 ± 20.34 mg/L vs. 3.20 ± 1.43 mg/L; *p* < 0.001) and an increase in erythrocyte sedimentation rate (ESR) (24.97 ± 11.54 mm/hr vs. 5.82 ± 1.83 mm/hr; *p* < 0.001). These findings highlight the heightened inflammatory state found in active autoimmune illness.

SLE patients had significantly higher levels of antinuclear antibodies (ANA) (78.33 ± 34.20 IU/mL) and anti-dsDNA antibodies (62.36 ± 31.09 IU/mL vs. 10.59 ± 5.14 IU/mL; *p* < 0.001). Both are well-known characteristics of SLE and are frequently utilized in diagnosis and disease monitoring. Anti-Smith antibodies were considerably higher in patients (16.04 ± 13.20 IU/mL vs. 6.58 ± 3.21 IU/mL; *p* < 0.001), validating the cohort’s autoimmune profile.

**Fig. 2:**
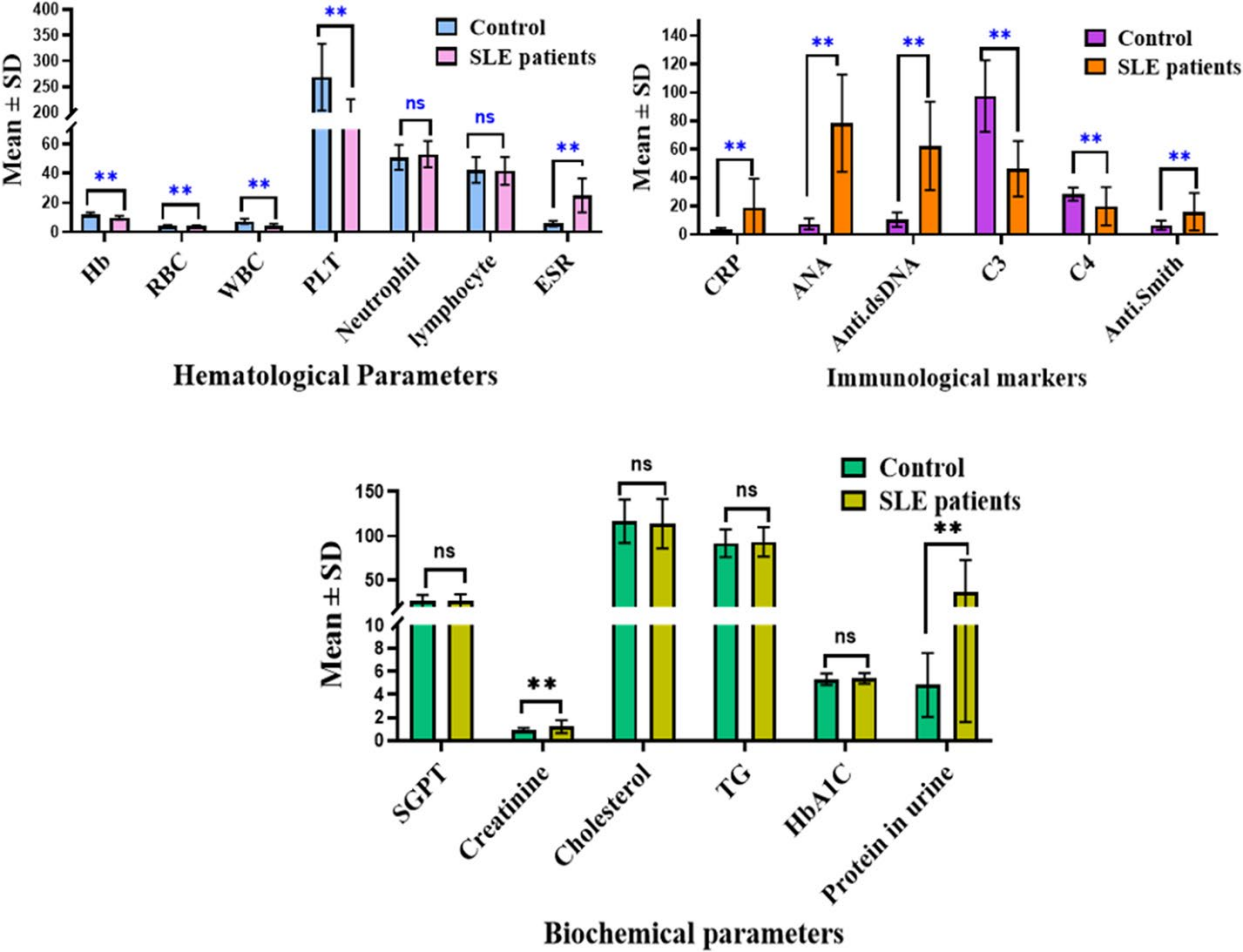
bar charts illustrate the mean difference values for hematological, immunological, and biochemical parameters between SLE patients and controls

SLE patients had significantly lower levels of complement proteins (mean C3 = 46.27 ± 19.49 mg/dL vs. 97.46 ± 25.22 mg/dL in controls; *p* < 0.001) and C4 = 19.94 ± 13.42 mg/dL vs. 28.43 ± 4.57 mg/dL (*p* < 0.001). This hypocomplementemia implies classical pathway activation and continuous immune complex-mediated tissue harm, which is consistent with active lupus. CRP, ESR, autoantibody titers, and miRNA expression showed non-normal distributions and were therefore analyzed using non-parametric methods. as indicated in Table 2 & Fig. 2.

**Table 2:** Comparison of Biochemical and Immunological Parameters between SLE Patients and Controls

Parameters	Controls (*n* = 50)	SLE Patients (*n* = 100)	t-test	*p*-value
	Mean ± SD			
Hemoglobin (g/dl)	12.15 ± 1.30	9.74 ± 1.26	10.91	< 0.001
RBC (10⁶/µL)	4.42 ± 0.43	4.22 ± 0.49	2.49	0.014
WBC (10³/µL)	7.41 ± 1.68	4.51 ± 1.10	12.67	< 0.001
Platelets (10³/µL)	268.64 ± 65.05	169.00 ± 56.87	9.64	< 0.001
Neutrophils (%)	50.81 ± 8.51	52.96 ± 9.06	−1.40	0.165
Lymphocytes (%)	42.27 ± 8.84	41.68 ± 9.49	0.37	0.713
CRP (mg/L)	3.20 ± 1.43	18.87 ± 20.34	−5.43	< 0.001
ESR (mm/hr)	5.82 ± 1.83	24.97 ± 11.54	−11.64	< 0.001
ANA (IU/mL)	7.42 ± 3.92	78.33 ± 34.20	−14.59	< 0.001
Anti-dsDNA (IU/mL)	10.59 ± 5.14	62.36 ± 31.09	−11.68	< 0.001
C3 (mg/dL)	97.46 ± 25.22	46.27 ± 19.49	13.71	< 0.001
C4 (mg/dL)	28.43 ± 4.57	19.94 ± 13.42	4.34	< 0.001
Anti-Smith (IU/mL)	6.58 ± 3.21	16.04 ± 13.20	−4.99	< 0.001
ALT (IU/L)	26.50 ± 7.30	27.16 ± 7.28	−0.52	0.602
Creatinine (mg/dL)	0.93 ± 0.16	1.22 ± 0.56	−3.60	< 0.001
Cholesterol (mg/dL)	116.80 ± 24.50	114.00 ± 27.93	0.60	0.548
Triglycerides (mg/dL)	92.10 ± 15.66	93.34 ± 16.63	−0.44	0.661
HbA1c (%)	5.34 ± 0.49	5.41 ± 0.46	−0.76	0.450
Protein in Urine (mg/dL)	4.83 ± 2.78	37.21 ± 35.60	−6.41	< 0.001

### MicroRNA Expression Analysis

**Fig. 3:**
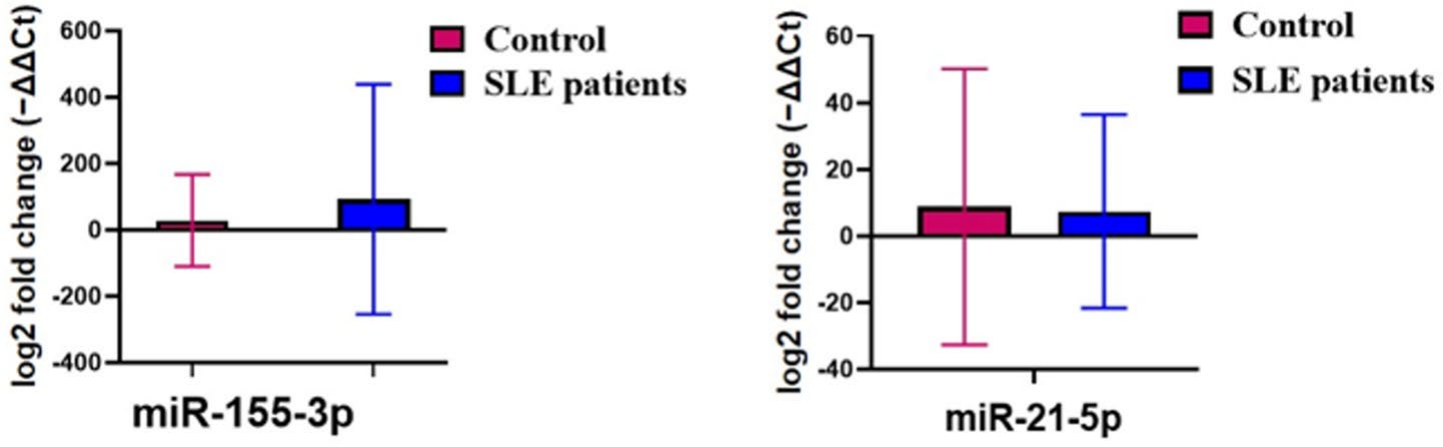
Expression levels are presented as log2 fold change (−ΔΔCt) relative to controls

Expression levels of microRNAs were analyzed to explore their potential role as novel biomarkers. miR-21-5p expression did not differ significantly between SLE patients and controls (median [IQR]: 2.83 [3.19–0.36] vs. 3.83 [4.12–0.29]; U = 2381.5, *p* = 0.637). In contrast, miR-155-3p expression was significantly higher in SLE patients compared with controls (median [IQR]: 29.94 [2.54–32.48] vs. 4.08 [4.15–0.07]; U = 935.5, *p* < 0.001), indicating an association with SLE-related immune activation. **(**Table 3; Fig. 3**).**

**Table 3:** Mann-Whitney U Test for Comparison of miRNA Expression Between SLE Patients (*n* = 100) and Controls (*n* = 50)

miRNA	Group	Median [Q1-Q3]	U Statistic	*p*-value
miR- 21-5p	Controls	0.78 (0.29–4.12)	2381.5	0.637
	SLE	0.96 (0.36–3.19)		
miR-155-3p	Controls	1.23 (0.07–4.15)	935.5	< 0.001
	SLE	10.53 (2.54–32.48)		

### Correlation Studies

Correlation analysis demonstrated that miR-21-5p was not significantly associated with any of the examined clinical, hematological, or immunological markers. In contrast, miR-155-3p exhibited noteworthy associations. It showed a significant positive correlation with anti-Smith antibody levels (ρ = 0.247, *p* = 0.013) and with serum creatinine (ρ = 0.237, *p* = 0.017), indicating a weak but statistically significant association with selected immunological and renal-related laboratory parameters. Additionally, a moderate correlation was observed between miR-155-3p and miR-21-5p (ρ = 0.472, *p* < 0.001), suggesting possible co-regulation or involvement in shared pathogenic pathways **(**Table 4**).**

**Table 4:** Spearman Correlations Between miRNAs and Key Clinical Parameters

Parameters	miR-21-5p		miR-155-3p	
	ρ	*p*-value	ρ	*p*-value
Hemoglobin (g/dl)	−0.043	0.668	−0.119	0.240
RBC (10⁶/µL)	0.030	0.771	0.098	0.332
WBC (10³/µL)	0.085	0.403	0.044	0.663
Platelets (10³/µL)	−0.116	0.250	−0.054	0.592
Neutrophils (%)	0.018	0.859	−0.004	0.970
Lymphocytes (%)	−0.035	0.729	−0.038	0.709
CRP (mg/L)	−0.097	0.337	0.160	0.112
ESR (mm/hr)	−0.041	0.685	0.042	0.677
ANA (IU/mL)	0.037	0.712	0.072	0.476
Anti-dsDNA (IU/mL)	−0.162	0.107	−0.122	0.225
C3 (mg/dL)	0.040	0.692	−0.066	0.514
C4 (mg/dL)	0.040	0.693	−0.004	0.966
Anti-Smith (IU/mL)	0.144	0.154	0.247*	0.013
ALT (IU/L)	0.019	0.855	−0.030	0.769
Creatinine (mg/dL)	−0.032	0.750	0.237*	0.017
Cholesterol (mg/dL)	−0.123	0.223	0.063	0.530
Triglycerides (mg/dL)	0.029	0.774	0.028	0.781
HbA1c (%)	−0.126	0.212	0.018	0.860
Protein in Urine (mg/dL)	−0.056	0.581	0.167	0.097
miR-155-3p	0.472**	< 0.001	1	-
miR-21-5p	1	-	0.472**	< 0.001

### miRNA expression levels among SLE patients with different disease activity levels

The Kruskal–Wallis’s test, which was applied to compare median ranks of miRNA expression levels among SLE patients with different disease activity categories (low, moderate, high, and severe). For miR-21-5p, mean ranks ranged from 49.23 in the low activity group to 54.20 in the moderate group, with no statistically significant difference among groups (H = 0.411, *p* = 0.938). Similarly, for miR-155-3p, mean ranks ranged between 44.00 (low activity) and 52.77 (high activity), with no significant group differences (H = 1.321, *p* = 0.724). Overall, no significant associations were observed between disease activity level and expression ranks of the assessed miRNAs. As shown in Table 5.

**Table 5:** Mean ranks and Kruskal–Wallis test results for miRNA expression levels among SLE patients with different disease activity levels

Disease Activity	miR-21-5p (Mean Rank)	miR-155-3p (Mean Rank)
Low	49.23	44.00
Moderate	54.20	52.53
High	49.77	52.77
Severe	49.58	50.90
**Kruskal–Wallis H**	0.411	1.321
**p-value**	0.938	0.724

### Diagnostic Performance (ROC Curve Analysis)

**Fig. 4:**
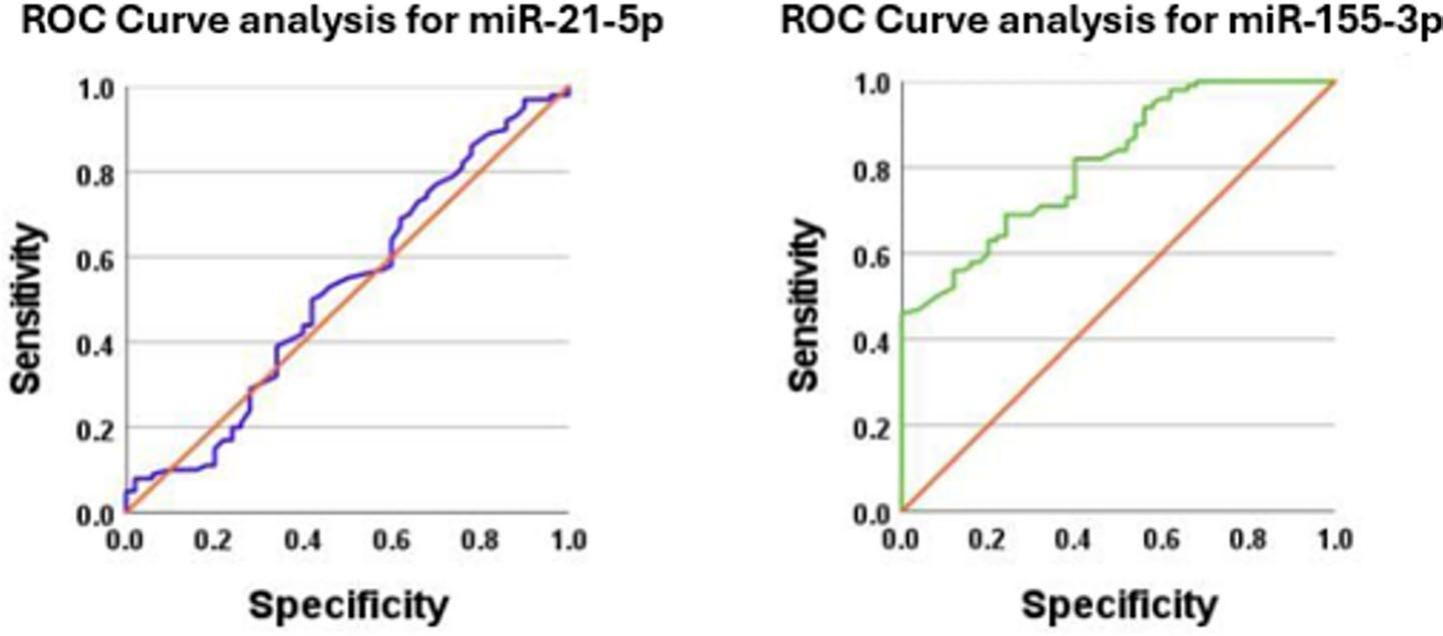
ROC curve for miRNAs among SLE patients

Receiver operating characteristic (ROC) curve analysis was conducted to assess the diagnostic potential of the microRNAs studied. miR-21-5p demonstrated poor discriminatory capacity between SLE patients and controls, with an AUC of 0.524 (95% CI: 0.422–0.625; *p* = 0.637), yielding only 10% sensitivity and 80% specificity at a cut-off of 4.9. In contrast, miR-155-3p demonstrated strong diagnostic ability, with an AUC of 0.813 (95% CI: 0.745–0.881; *p* < 0.001), very high sensitivity (99%), and moderate specificity (32%) at a cut-off of 40. Given the excellent diagnostic performance of established serological markers in this case–control setting, the present analysis does not allow assessment of incremental diagnostic benefit provided by miR-155-3p. **(**Table 6; Fig. 4**).**

**Table 6:** ROC analysis for miRNAs among the SLE patients

miRNA	AUC	Cut-off Value	Sensitivity	Specificity	*p*-value	95% CI (Lower-Upper)
miR-21-5p	0.524	4.9	10%	80%	0.637	0.422–0.625
miR-155-3p	0.813	40	99.0%	32.0%	< 0.001	0.745–0.881

**Fig. 5:**
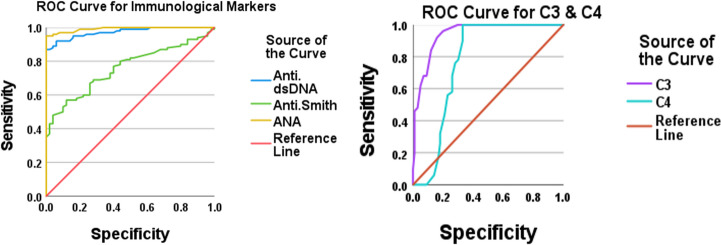
ROC curve for Immunological Markers among the SLE patients

For comparison, conventional immunological markers were also assessed. ANA exhibited outstanding diagnostic performance (AUC = 0.992, 95% CI: 0.983–1; *p* < 0.001), with 95% sensitivity and 100% specificity at a cut-off of 17 IU/mL. Anti-dsDNA also showed excellent diagnostic capacity (AUC = 0.973, 95% CI: 0.925–0.994; *p* < 0.001), achieving 87% sensitivity and 100% specificity at a cut-off of 22 IU/mL. Anti-Smith antibodies demonstrated good accuracy (AUC = 0.759, 95% CI: 0.684–0.843; *p* < 0.001) at a cut-off of 10.4 IU/mL, ROC analyses for complement markers were interpreted considering that lower values indicate disease presence. These ROC estimates reflect discrimination between established SLE cases and healthy controls and may therefore represent idealized performance compared with real-world diagnostic settings. **(**Table 7; Fig. 5**).**

**Table 7:** ROC analysis for Immunological Markers among the SLE patients

miRNA	AUC	Cut-off Value	Sensitivity	Specificity	*p*-value	95% CI (Lower-Upper)
Anti-Smith	0.759	10.4	75%	88%	< 0.001	0.684–0.843
Anti-dsDNA	0.973	22	87%	100%	< 0.001	0.925–0.994
ANA	0.992	17	95%	100%	< 0.001	0.983–1
C3	0.942	65	92%	84%	< 0.001	0.908–0.976
C4	0.771	20.9	100%	67%	< 0.001	0.695–0.846

## Discussion

The current study examined the biochemical, immunological, and molecular profiles of patients with systemic lupus erythematosus (SLE) in comparison to healthy controls, with a particular emphasis on whole-blood miRNA expression as exploratory biomarkers associated with SLE-related laboratory features. Importantly, the present work is a case–control cross-sectional study using healthy controls. This design may overestimate discriminatory performance and does not reflect real-world diagnostic uncertainty, where SLE must be differentiated from other autoimmune/inflammatory mimicking conditions. Therefore, the ROC findings should be interpreted as exploratory and require validation in prospective diagnostic accuracy cohorts, including disease controls [17].

The findings not only confirm well-known hematological and immunological changes in SLE but also offer new insights into the diagnostic performance of specific miRNAs, specifically miR-155-3p. From a hematological viewpoint, the observed reductions in hemoglobin, red blood cell (RBC) count, white blood cell (WBC) count, and platelet levels in SLE patients are consistent with previous reports describing SLE hematologic manifestations, which can be attributed to autoimmune-mediated destruction, bone marrow suppression, or chronic inflammation [18].

Inflammation markers, such as CRP and ESR, rise in response to this disease, suggesting that the body is certainly undergoing significant inflammation. Prior research has repeatedly found this pattern. Additionally, immune system indicators such as anti-dsDNA antibodies and ANA levels are typically greater in SLE patients. At the same time, during periods of disease deterioration, their levels of C3 and C4 complement decline, indicating that their immune system is working overtime against the disease. It’s related to how the immune system experiences a state of hyperactivation during active phases [19].

Increased levels of creatinine and protein in the urine of these people are indicative of renal disease. This is consistent with our prior knowledge that lupus nephritis is a particularly challenging component of systemic lupus erythematosus. What’s remarkable is that additional parameters like lipid profile, white blood cell count, liver enzymes, and blood sugar levels remained relatively unchanged. It could be due to the fact that lupus affects people differently, the efficacy of the medications, or the fact that these individuals did not have any other significant health issues that would have impacted the results [20].

It is important to note that serum creatinine is a limited surrogate marker for lupus nephritis activity and may be influenced by multiple confounding factors, including baseline renal function, hydration status, medication exposure, and chronic renal damage. Therefore, the observed association between miR-155-3p and creatinine should be interpreted cautiously and does not reflect active renal inflammation. miR-155-3p showed weak but significant associations with renal-related laboratory parameters, particularly serum creatinine. Given the absence of standard nephrology indices (e.g., eGFR, uPCR/uACR, or 24-hour proteinuria), these findings should not be interpreted as evidence of active renal involvement or lupus nephritis [21].

A molecular analysis of circulating miRNAs identified several upregulated patterns. Because it has a very strong positive relationship with anti-Smith antibodies and creatinine levels and is much more common in SLE patients, miR-155-3p may be associated with immune activation and renal-related laboratory changes, without implying direct renal injury or disease activity.

These findings align with prior studies indicating that miR-155 facilitates inflammatory mechanisms, encompassing immune cell activation and cytokine production [22].

Although most studies have focused on miR-155-5p, emerging evidence indicates that the −3p strand is also biologically active and may reflect immune cell–specific regulatory processes. Given the use of whole-blood samples in the present study, miR-155-3p was selected to explore its association with immune activation and laboratory features relevant to SLE [23].

miR-21-5p levels were not significantly different between groups, contradicting a prior study that suggested it to be elevated in autoimmune diseases. This variance may be related to variations in patient demographics, and disease activity distribution [24].

The observed differences in miR-21-5p expression highlight the underlying heterogeneity of SLE. Differences in ethnicity, sample size, disease duration, and treatment regimens between studies may explain some of the inconsistent results. Furthermore, methodological variables such as sample type (serum vs. plasma), normalization procedures, and analytical platforms may have altered miR-21-5p detection. This emphasizes the significance of standardizing miRNA detection methodologies to improve comparability between research and establish their clinical relevance [25].

The ROC curve provided supplementary insights into the diagnostic efficacy of these markers. Among the miRNAs, miR-155-3p exhibited the highest diagnostic accuracy (AUC = 0.813), Although miR-155-3p demonstrated moderate discriminatory performance, its specificity was limited and its added diagnostic value beyond conventional markers could not be established in the present study. Therefore, miR-155-3p should be regarded as an exploratory complementary marker rather than a replacement or proven enhancer of existing diagnostic assays. Conversely, miR-21-5p exhibited minimal diagnostic utility, aligning with the absence of notable differential expression. In comparison to conventional immunological markers, miRNAs have inferior diagnostic capability. Both ANA and anti-dsDNA exhibited excellent accuracy (AUC > 0.97) along with elevated sensitivity and specificity, confirming their recognized position as gold-standard laboratory markers for SLE diagnosis [26].

An important limitation of the present study is the absence of disease-control groups. The comparison with healthy controls alone does not capture the clinical diagnostic challenge of distinguishing SLE from other autoimmune, inflammatory, or infectious conditions. Consequently, the diagnostic accuracy of both conventional biomarkers (e.g., ANA, anti-dsDNA) and miRNAs may be overestimated. Future studies incorporating relevant disease controls are required to validate these findings in clinically meaningful diagnostic contexts [27].

Given the excellent diagnostic performance of established serological markers such as ANA and anti-dsDNA in this case–control setting, demonstrating incremental diagnostic value of additional biomarkers is inherently limited. Therefore, the present findings should be interpreted as exploratory, supporting a complementary rather than additive diagnostic role for miR-155-3p [28].

Although miR-155-3p has shown potential sensitivity, its low specificity raises questions about its usage as a solo diagnostic marker. However, in clinical practice, biomarkers are rarely used in isolation. Instead, including miRNAs into multiplex panels that include serological, immunological, and biochemical markers may help increase diagnostic accuracy and identify patients with unusual symptoms. Such integrative techniques may potentially allow early diagnosis and better monitoring of disease activity [29].

Anti-Smith antibodies exhibited satisfactory accuracy (AUC = 0.759), rendering them a valuable adjunct marker [30]. The data suggest that although certain miRNAs, especially miR-155-3p, exhibit potential as diagnostic instruments, they are unlikely to supplant established immunological assays in contemporary clinical practice. Conversely, integrating them into multi-marker panels may enhance diagnostic sensitivity, especially in early or atypical instances [31].

Our data revealed a moderate, very significant positive association between miR-155-3p and miR-21-5p (ρ = 0.472, *p* < 0.001), indicating co-regulated expression or convergent involvement in SLE pathogenesis. Both microRNAs have been connected to immune cell activation and inflammatory signaling; miR-155-3p is highly associated with B-cell activation and pro-inflammatory responses, whilst miR-21 regulates T-cell phenotypes and modulates apoptosis and fibrosis pathways. From a molecular standpoint, the interaction between miR-155 and miR-21 may reflect a larger disruption of immunological homeostasis in SLE. While miR-155 promotes pro-inflammatory pathways, miR-21 helps with tissue remodeling and fibrosis. Their co-expression may thereby hasten organ damage, notably in the renal and cardiovascular systems, which are important causes of morbidity and mortality in lupus patients. These findings enhance the case for examining miRNAs not only as indicators, but also as possible therapeutic targets [32].

This study’s findings indicate that specific miRNAs are upregulated in SLE and correlate with significant immunological and renal characteristics, suggesting their potential as biomarkers for disease activity and organ involvement. The observed heterogeneity in expression, along with the insufficient specificity of the most effective miRNA, underscores the necessity for further research to corroborate these findings and explore their use alongside conventional markers. Longitudinal studies investigating miRNA alterations during the disease progression and in reaction to treatment may elucidate their predictive and therapeutic value [33].

miRNA expression in the present study was measured from whole blood rather than plasma or serum. Whole-blood miRNA profiles may be influenced by cellular components, including leukocytes, platelets, and erythrocytes, as well as by hemolysis. Consequently, direct comparison with studies assessing circulating (plasma/serum-derived or exosomal) miRNAs should be made with caution. While whole-blood miRNA expression may reflect immune cell activity relevant to SLE pathogenesis, this methodological difference represents a limitation for biomarker translation and warrants further validation using standardized circulating miRNA platforms [34].

An important limitation of the present study is its case–control design comparing established SLE patients with healthy controls. While this approach is suitable for exploratory biomarker evaluation, it tends to overestimate diagnostic performance and does not reflect real-world diagnostic uncertainty, where SLE must be distinguished from other autoimmune, inflammatory, or infectious conditions with overlapping clinical features. As disease-control groups were not included, the present findings should be interpreted as hypothesis-generating. Future studies incorporating clinically relevant disease controls and prospective diagnostic cohorts are required to validate the clinical utility of miR-155-3p [35].

Future research should concentrate on multicenter studies with bigger and more diverse cohorts to confirm the repeatability of these findings. Estimated glomerular filtration rate (eGFR) and standardized proteinuria indices (uPCR/uACR or 24-hour protein) were not available, which limits the ability to assess renal disease severity or activity. In parallel, experimental studies are needed to elucidate the molecular involvement of miRNAs in lupus pathogenesis. Importantly, advancements in miRNA-based medicines, such as antagomiRs and miRNA mimics, may open the way for new targeted therapy in SLE. The incorporation of miRNA profiling into standard clinical practice may potentially improve individualized care, influencing both diagnosis and therapeutic options.

## Conclusion

This study found significant hematological, biochemical, and immunological changes in people with systemic lupus erythematosus compared to healthy persons, indicating the disease’s underlying inflammatory and autoimmune processes. Traditional serological indicators, such as ANA, anti-dsDNA, and anti-Smith antibodies, showed great diagnostic accuracy, while complement levels (C3, C4) offered additional information about disease activity. Notably, miRNA-155-3p expression was significantly higher in SLE patients, associated with selected renal-related laboratory parameters, without establishing renal injury or disease activity., may serve as a complementary biomarker alongside established serological markers, in contrast, miR-21-5p showed no substantial diagnostic significance. Overall, miR-155-3p showed exploratory discriminatory ability and associations with selected immunological and renal-related laboratory parameters, including serum creatinine. These findings should be interpreted cautiously, as standard nephrology indices were not available, and further prospective studies are required before clinical translation.

## Data Availability

All data generated or analyzed during this study are included in this article.
